# Increases in Great Lake winds and extreme events facilitate interbasin coupling and reduce water quality in Lake Erie

**DOI:** 10.1038/s41598-021-84961-9

**Published:** 2021-03-11

**Authors:** Aidin Jabbari, Josef D. Ackerman, Leon Boegman, Yingming Zhao

**Affiliations:** 1 Physical Ecology Laboratory, Department of Integrative Biology, University of Guelph, Guelph, ON Canada; 2 Environmental Fluid Dynamics Laboratory, Department of Civil Engineering, Queen’s University, Kingston, ON Canada; 3 Ontario Ministry of Natural Resources and Forestry, Aquatic Research and Monitoring Section, Wheatley, ON Canada; 4 Present Address: Biogeochemistry and Earth System Modelling, Department of Geoscience, Environment and Society, Université Libre de Bruxelles, Brussels, Belgium

**Keywords:** Limnology, Climate change, Physical oceanography, Freshwater ecology

## Abstract

Climate change affects physical and biogeochemical processes in lakes. We show significant increases in surface-water temperature (~ 0.5 °C decade^−1^; > 0.2% year^−1^) and wave power (> 1% year^−1^; the transport of energy by waves) associated with atmospheric phenomena (Atlantic Multidecadal Oscillation and Multivariate El Niño/Southern Oscillation) in the month of August between 1980 and 2018 in the Laurentian Great Lakes. A pattern in wave power, in response to extreme winds, was identified as a proxy to predict interbasin coupling in Lake Erie. This involved the upwelling of cold and hypoxic (dissolved oxygen < 2 mg L^−1^) hypolimnetic water containing high total phosphorus concentration from the seasonally stratified central basin into the normally well-mixed western basin opposite to the eastward flow. Analysis of historical records indicate that hypoxic events due to interbasin exchange have increased in the western basin over the last four decades (43% in the last 10 years) thus affecting the water quality of the one of the world’s largest freshwater sources and fisheries.

## Introduction

Climate change has increased water temperature and altered wind-driven water movements in aquatic systems^1,2^. This applies not only to the mean conditions^3,4^, but also to the frequency of extreme events (i.e., near the upper ends of the range of observed values^5^, > 80th percentile). For example, high air temperature or powerful winds^5–9^ has affected the behaviour of surface gravity waves^10^. Understanding the changes in wind and wave climate provides insight into the prediction and management of climate change impacts related to coastal dynamics, such as coastal erosion and sediment budgets, water motions, and biological responses^6,11,12^. Several studies on the impacts of climate change on oceanic waves^12–15^ have been undertaken, including a recent study^16^ that shows a 0.41% annual increase in global wave power (*WP*; the transport of energy by waves, which represents the temporal variations of energy transferred from the atmosphere to the ocean surface motion over cumulative periods of time^16,17^ (Eq. 2) due to stronger winds caused by increases in sea surface temperature. The oceanic wave climate also responds to global atmospheric phenomena (e.g., El Niño Southern Oscillation and the Atlantic Multidecadal Oscillation), in which sea surface temperature modifies wind patterns and storm cyclogenesis^12,18–20^. A systematic long-term assessment of climate warming impacts on waves in lakes remains to be undertaken, but should include winds, which are one of the principal sources of mechanical energy for lake circulation and interbasin coupling (e.g., exchange)^21–24^.

**Figure 1:**
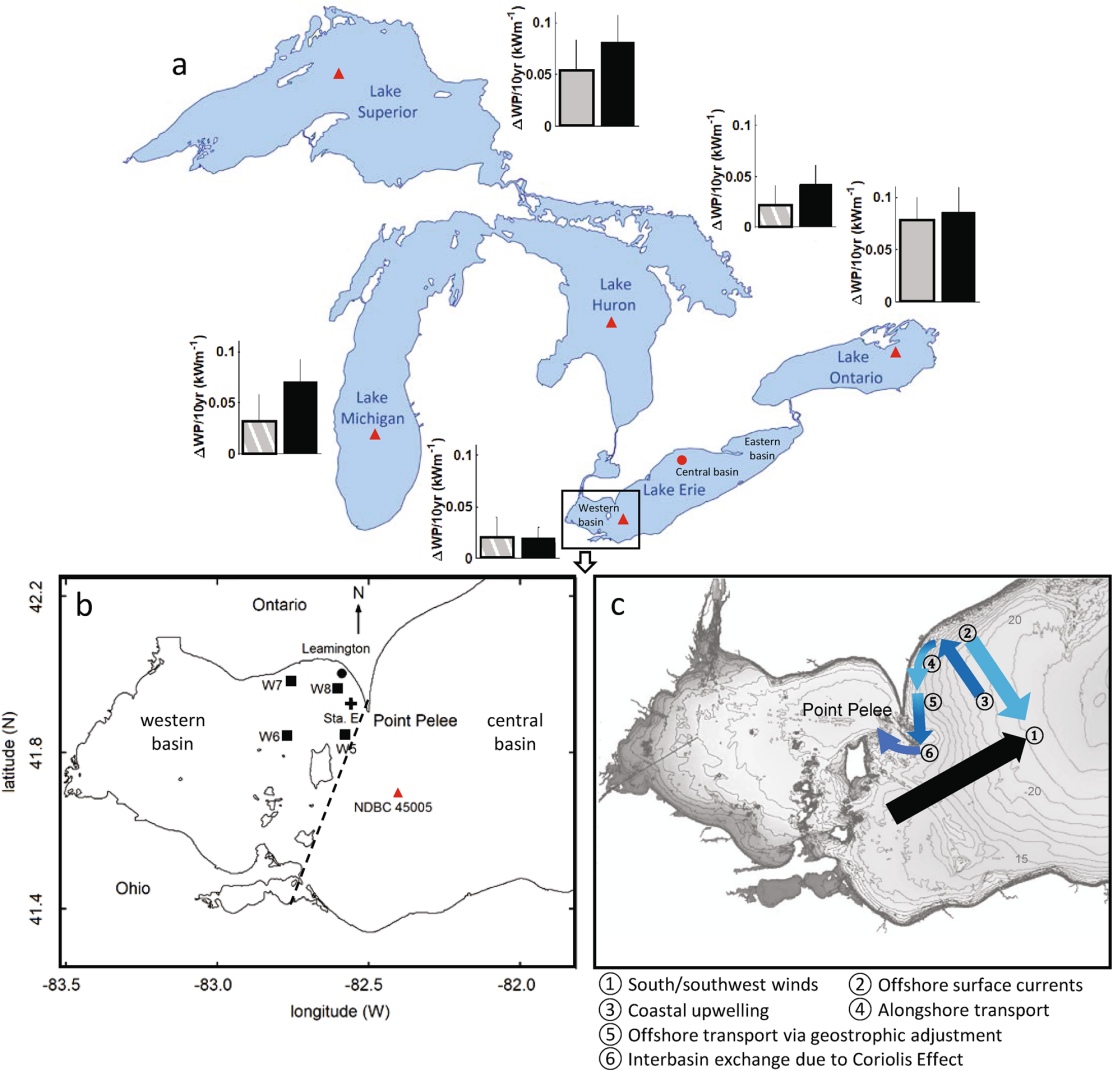
Map of Great Lakes and stations in this study. (**a**) Map of the Laurentian Great Lakes with the location of weather and wave buoys (red triangles) and the location of the western basin indicated in the outline. The grey and black bars show the rate of increase in wave power during August (grey) and August during winds from the south and southwest direction (black) between 1980 and 2018 (Supplementary Table 1). The length of the error bars shows the standard error. The hatched grey bars indicate relationships that are not statistically significant (i.e., p > 0.05); all the black bars are significant (i.e., p < 0.05). The red circle in Lake Erie is the location of the weather and wave buoys near Port Stanley (Supplementary Table 1). (**b**) Western basin of Lake Erie with the location of the bottom water temperature (*LBT*), dissolved oxygen (*DO*), and water current measurements in 2017^22^ and 2018 (indicated by + symbol; Sta. E: 41.9398^∘^N, 82.5527^∘^W). The red triangle (41.677°N, 82.398°W; 9.8 m depth) is the weather and wave Sta. NDBC 45005. The circle (42.01567°N, 82.58185°W; 4.1 m depth in Leamington, ON) is the location of the water temperature time series measurements (1998–2018; no data for 2012) from the Ontario Ministry of Natural Resources and Forestry (MNRF) and the squares are the locations of limnological records from biweekly sampling cruises (bottom dissolved oxygen 2007–2018; total phosphorus 2000–2018) conducted in August by MNRF (W5: 41.8832°N, 82.613°W, W6: 41.8545°N, 82.7623°W, W7: 41.9907°N, 82.7633^∘^W, and W8: 41.9865°N, 82.5758°W). The dashed line in (**b**) indicates the boundary between the western and central basins. See Supplementary Table 2 for details of station locations and instruments used. (**c**) Schematic of the mechanism of upwelling and intrusion of hypolimnetic water from the central basin to the western basin. Winds from the south and southwest direction cause offshore surface currents (light blue) due to Ekman transport that result in coastal upwelling of hypolimnetic water (dark blue), which is entrained in the alongshore surface current. Following relaxation of the winds, the upwelled water sinks due to the pressure gradient and intrudes into the western basin due to the Coriolis effect (e.g., geostrophic adjustment). All maps were obtained from the National Centers for Environmental Information (NCEI), National Oceanographic and Atmospheric Administration (NOAA) (https://www.ngdc.noaa.gov/mgg/greatlakes/). (**c**) is modified from https://www.ngdc.noaa.gov/mgg/greatlakes/erie.html with bathymetry contours provided in metres.

The Laurentian Great Lakes, which consist of lakes Superior, Michigan, Huron, Erie, and Ontario (Fig. 1a), are the largest group of freshwater lakes on Earth; they contain 21% of the world's volume of fresh surface water. These lakes have been affected by climate change in several ways including increased surface water temperature, longer summer stratification related hypoxia (i.e., dissolved oxygen [DO] concentrations < 2 mg L^−1^ observed in Lake Erie, Lake Superior (Green Bay) and Lake Michigan (Saginaw Bay)) as well as increased occurrence of weather extremes and harmful algal blooms (HAB), which degrade water quality^3,7,25,26^. Lake Erie is the shallowest (average depth ~ 19 m) of the Great Lakes, has the shortest residence time (~ 3 years)^7^, and yet its watershed is home to one-third of the total human population of the Great Lakes basin^21^. Not surprisingly, it has a long history of eutrophication and hypoxia related to urbanization, industry, and agriculture^25,26^. For example, the largest HAB recorded in Lake Erie occurred in 2015 when the algal bloom spread ~ 200 kms across most of the lake^27^. Moreover, during a previous HAB in 2014, half a million people living around the southwestern basin of the lake suffered a domestic water use ban due to hepatotoxins produced by the cyanobacterium *Microcystis*^28^.

Lake Erie is not uniform spatially, rather it consists of three interconnected basins (Fig. 1a) with dominant wind forcing from synoptic high winds (5–10 days period) and diurnal sea breeze in the coastal and shallow regions during summer^29–31^. Thermal structure in the basins are different in the summertime. The relatively shallow western basin thermally stratifies diurnally or for short periods of calm and warm atmospheric conditions (~ 4.5 days) that can result in hypoxia^30,32^ due to oxygen depletion from the mineralization of organic matter in the hypolimnion and within the sediment. The deeper central and eastern basins stratify seasonally^21,33,34^, which in the former has increased in spatial extent since the mid-1990s; in recent times, this has been primarily due to increased dissolved reactive phosphorus loads from agriculture^35^. Hypoxia, especially in the western basin, can have significant negative impacts on fish distributions^36,37^ and benthic invertebrates including burrowing mayflies in the genus *Hexagenia*^38,39^. Whereas the eastward hydraulic flow from Detroit River into the central basin is the dominant flow direction in the western basin, the intrusion of hypolimnetic water from the central basin from south of Point Pelee (Fig. 1b) can cause episodic stratification in the western basin during August^22,40,41^. This occurs when high winds (> 8 m s^−1^) from the south and southwest that are the common wind directions over the Great Lakes^23^, tilt the thermocline upward in the western and northern part of the central basin due to Ekman transport of surface water southward^22,38,42–45^. As this hypolimnetic water upwells into shallower depths it can be transported counter clockwise by the alongshore surface currents moving to the west^32^. If there is a calm period following the high winds, the upwelled water in the northwestern part of the central basin will flow southward because of the pressure gradient and also in a clockwise direction (to the west) because of the Coriolis effect, and so will intrude into the western basin (i.e., a geostrophic flow) opposite to the hydraulic flow from the Detroit River (Fig. 1c)^22,32,46^. This causes the rapid (on the order of hours) formation of a thermocline within the northeastern portion of the western basin (Pelee Passage) due to the intrusion of low temperature bottom water^22,42^, which can also be hypoxic^22^ or anoxic (i.e., DO $\approx$ 0) at the sediment surface^22^ and contain high soluble reactive phosphorus concentrations (SRP; 0.02–0.05 mg L^−1^)^47–49^. Low values of sediment oxygen uptake are observed during these events in the western basin due to stratification and weak bottom shear and turbulence, which results in thicker diffusive sublayer^22^.

Interbasin exchange has been observed in lakes with multiple basins elsewhere (e.g., Lake Geneva^50^, Nechako Reservoir^51^) as well as in the Great Lakes region (e.g., Muskegon Bay^52^, Green Bay^53^, Kempenfelt Bay^54^, Pere Marquette River^55^)*.* In Lake Michigan, for example, high winds can lead to coastal upwelling into Muskegon Lake causing episodic hypoxia^52^. In case of Lake Erie, interbasin exchange was identified as the dominant cause (63%) of hypoxia in the northeastern portion of the western basin during biweekly fishing trawls in August over the past 30 years^22^. However, there are no long-term continuous water quality observations to assess the occurrence and historic trends in these hypoxic events. Extreme winds prevailing from upwelling favourable directions (i.e., from the south and southwest) can generate strong surface waves and water currents through momentum flux at the air–water interface. Therefore, *WP* can be used as an indicator or proxy (but not the cause) of interbasin exchange. Here, we examine the historical trends in water temperature, winds and resultant waves in the context of climate change in the summer in the Great Lakes (Fig. 1a) with an emphasis on the western basin of Lake Erie (Fig. 1b). We examine data for August, which is the month when hypoxia is most likely to occur in dimictic north temperate lakes before the fall turnover, and when large HAB have been observed in the western basin of Lake Erie. August is also the time when the spatial extent of hypoxia in the central basin is the largest and when the aforementioned upwelling into the western basin is likely to occur^22,40,56^. The data examined are from buoys with the longest historical records (Fig. 1a and Table S1). We examine winds from the south and southwest directions, which are the common wind directions over the Great Lakes during August, and which are favourable for upwelling into the western basin of Lake Erie. The results show that the *WP* in Great Lakes has increased in the past 40 years. A pattern in *WP* (a proxy for hypoxic upwelling events into the western basin of Lake Erie) has also increased in frequency over this time, which has implications for the water quality (e.g., dissolved oxygen and total phosphorus) of the lake. The increased frequency of interbasin upwelling was confirmed using historical records of lake bottom water temperature (*LBT*), as well as dissolved oxygen and total phosphorus concentrations. This is the first time that *WP* has been identified as an indicator of climate change-driven biogeochemical responses in lakes.

### Long-term trends in *WP* and *LST* in the Great Lakes

First, we investigate the historical trends in average lake surface temperature (*LST*), wind, and waves in the Great Lakes during August. Results show that *LST* and *LST*_*w*_ (hereinafter subscript ''*w*'' is used to denote the variables measured during upwelling favourable winds from 180° to 270°, clockwise from north) have both increased significantly (p < 0.05) by ~ 0.5 °C decade^−1^ (> 0.2% year^−1^) since 1980, although lower trends were observed in lake Erie and Michigan (Figs. S1–S5 ((a) and (b)) and Table S1). These changes in the *LST* correspond to a warming trend in air temperature (*Tair*); the average *Tair* over the Great Lakes increased significantly by ~ 0.4 $\pm$ 0.2 ($\pm$ standard error) ^o^C decade^−1^ since 1980 (Fig. S6a,b). There was an associated significant increase in wind speed (*W*) over the Great Lakes during August (*W*_*w*_) of ~ 0.4 $\pm$ 0.1 m s^−1^ decade^−1^ for winds from the south and southwest (Figs. S1–S5 ((c) and (d)) and Table S1). Consequently, the wind stress associated with wind from the south and southwest over the water surface of the Great Lakes (${\tau }_{w}=0.0012{\rho }_{air}{W}_{w}^{2}$, where ${\rho }_{air}$=1.22 kg m^−3^ is the density of air^57^, and the wind speed is measured 10 m above the water) increased significantly by 0.006 $\pm$ 0.002 Pa decade^−1^ during August (3.0 $\pm$ 0.9% year^−1^; Figs. S1–S5 ((e) and (f)) and Table S1).

**Figure 2:**
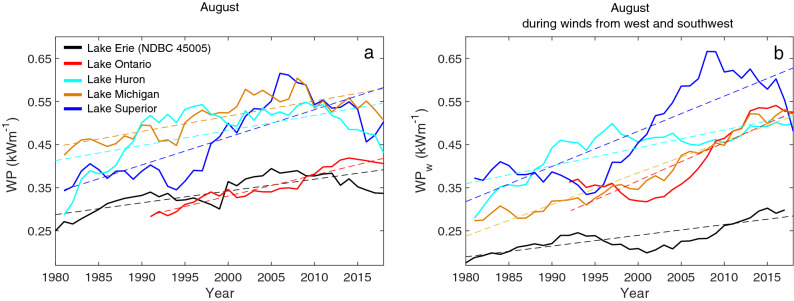
Historical patterns in wave power in Great Lakes. 10 year moving average of wave power (*WP*) during the August (**a**) and during August with the wind from south and southwest and (*WP*_*w*_; **b**). The dashed lines show the linear regression (statistical results provided in Table S1).

The effects of increased wind stress can also be seen in wave power, which is a function of the square of significant wave height (the mean value of the largest third of the wave heights during typically 1 h, *SWH*) and the wave period (${T}_{p}$; i.e., $WP \propto {{T}_{p} \times SWH}^{2}$); and changes in wind are reflected in wave power ($WP \propto {W}^{2.4}$ and $\propto {W}^{5}$ for developing and fully developed waves, respectively; see “Materials and methods”). The average *SWH* and *SWH*_*w*_ in the Great Lakes during August have increased significantly by 0.03 $\pm$ 0.02 and 0.04 $\pm$ 0.03 m decade^−1^, respectively (i.e., ~ 1.0 $\pm$ 0.8% and ~ 1.7 $\pm$ 1.5% year^−1^, respectively), and this is largely driven by the increase in the frequency of extreme surface winds^58^ (Figs. S1–S5g and h; *WP* responds to changes in mean values, but it is more sensitive to extreme events because *WP* $\propto { SWH}^{2}$^16^). Consequently, the average *WP* and *WP*_*w*_ in the Great Lakes during August have increased by ~ 0.04 $\pm$ 0.02 and ~ 0.06 $\pm$ 0.03 kW m^−1^ decade^−1^, respectively (i.e., ~ 1.0 $\pm$ 0.6% and ~ 2.0 $\pm$ 0.9% year^−1^, respectively; Fig. 2). In Lake Erie, *WP*_*w*_ during August increased significantly by 0.02 $\pm$ 0.01 kW m^−1^ decade^−1^ (1.4 $\pm$ 0.2% year^−1^; Fig. 2 and Table S1; the increasing trend in *WP* = 0.02 $\pm$ 0.02 or 0.5 $\pm$ 0.1% was not statistically significant). It is relevant to note that these results are based on observations from a single buoy per lake; the one with the longest available data records (Fig. 1a and Table S2). However, the wind records and historical wave trends between buoys Sta. NDBC 45005 and Port Stanley in Lake Erie (Fig. 1a), which are ~ 130 km apart, are consistent based on the available records. Specifically, wind speed and direction in 2018 have Pearson correlation coefficients, *r* > 0.6 (Fig. S7a,b, respectively); *W*_*w*_ and *WP*_*w*_ are also correlated with *r* = 0.51 and 0.67, respectively, during August of 1990–2018 and the buoys show similar temporal increases in *WP*_*w*_ (~ 0.025 $\pm$ 0.02 and 0.02 ± 0.01 kW m^−1^ decade^−1^ in Port Stanley and Sta. NDBC 45005, respectively). The trends in historical *LST*_*w*_ and *WP*_*w*_ are related statistically (i.e., higher mutual information; Fig. S8) similar to the relationship described for global sea surface temperature and oceanic *WP* used as an indicator of climate change^16^.

The long-term variations in *WP* and *LST* may be related to the global atmospheric phenomena. The *LST*_*w*_ anomaly in all the lakes show an increasing trend beginning in 1995 (Fig. S9a), which corresponds to the switch from the negative mode of the Atlantic Multidecadal Oscillation (AMO) to the positive mode (associated with increased tropical cyclone activity and stronger westerly winds) between the 1980s and the early 2000s (Fig. S9b)^16^. Both the *WP*_*w*_ and *LST*_*w*_ anomaly are positively correlated with the AMO (*r* ~ 0.50 and ~ 0.55, respectively, since 1990). Similar to global oceanic wave power^16^, peaks in *WP*_*w*_ in the Great Lakes are associated with strong El Niño years (i.e., Multivariate El Niño/Southern Oscillation (MEI) greater than 1.5; Fig. S9c,d), which can contribute to the enhanced wind energy due to increased cyclonic events^16^. MEI and *WP*_*w*_ in Great Lakes are generally correlated by *r* > 0.45 since 1990, however, the impacts of global atmospheric events on temperature and water dynamics of Great Lakes requires further study.

### Episodic hypoxic upwelling events in the western basin of Lake Erie

**Figure 3:**
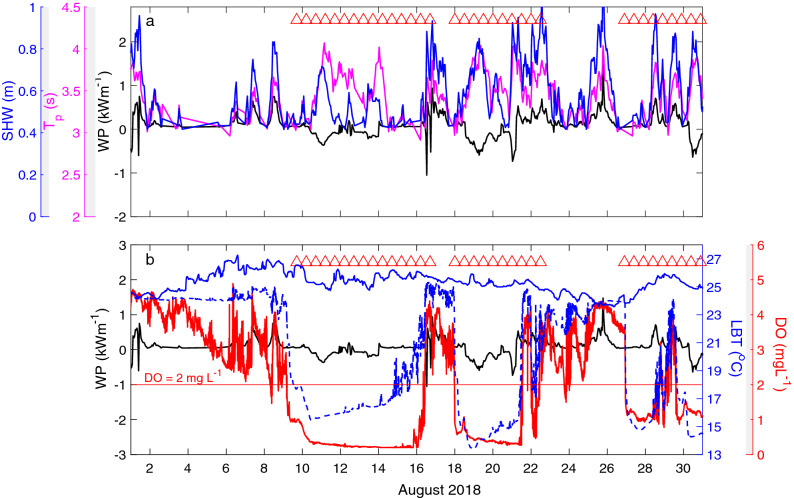
Wave power and bottom water temperature during August 2018 in the western basin of Lake Erie. (**a**) Time series of wave power (*WP*; black line), wave period (*T*_*p*_; magenta), and significant wave height (*SWH*; blue) recorded at Sta. NDBC 45005. (**b**) Time series of dissolved oxygen (DO; red) and water temperature (*LBT*; blue dashed-line) in Sta. E at 1 m above the bed and bottom water temperature in Leamington (blue solid-line) in August 2018. The red triangles represent the observed hypoxic events in the western basin of Lake Erie. The wave power of the waves from south and southwest (i.e., favourable for upwelling) are positive preceding upwelling.

**Figure 4:**
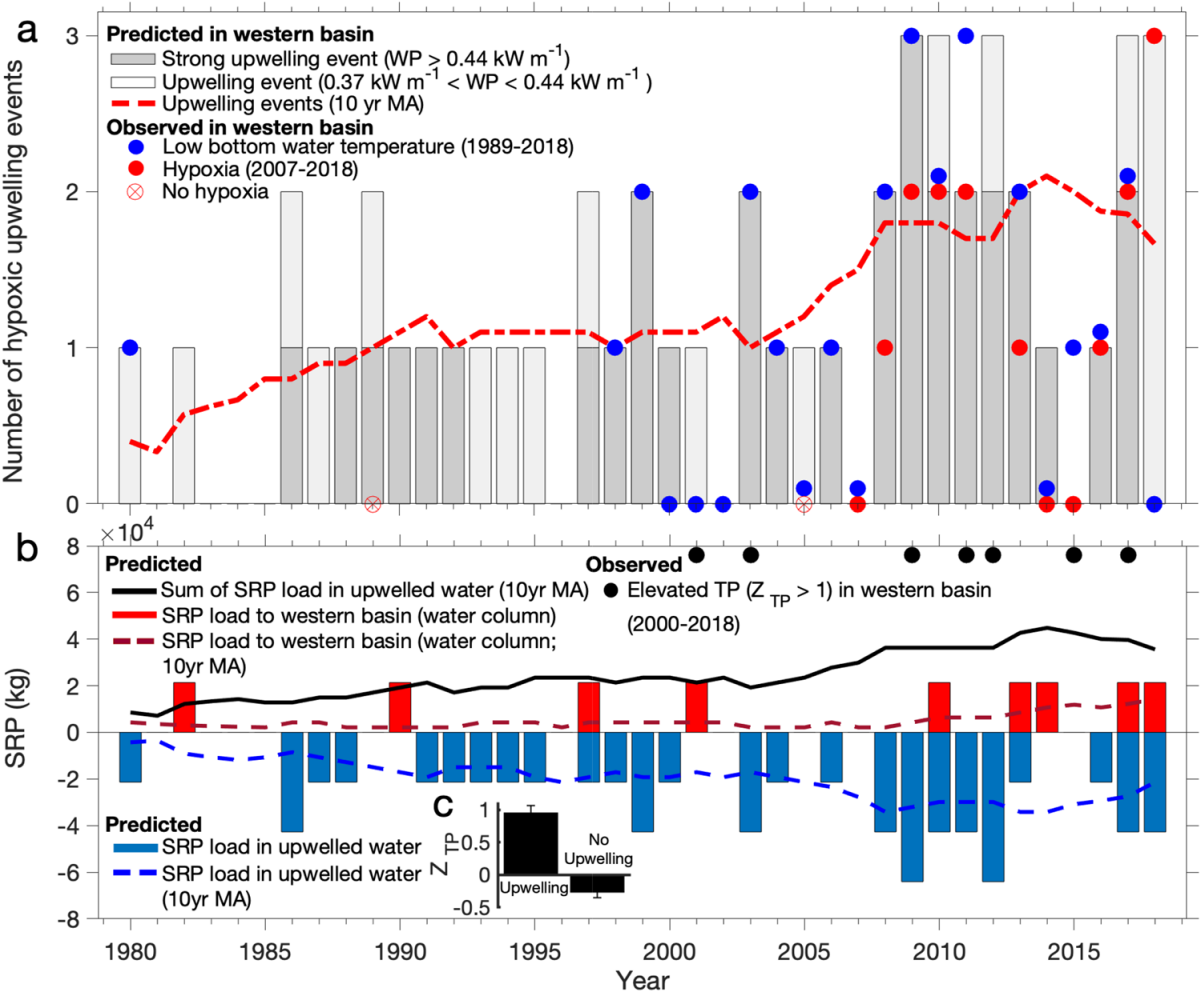
Number of hypoxic upwelling events in the western basin. (**a**) The number of hypoxic upwelling events based on patterns in wave power at Sta. NDBC 45005 (dark grey: average *WP*_*w*_ > 0.44 kW m^−1^, light grey: 0.37 < *WP*_*w*_ < 0.44 kW m^−1^), and observed low bottom water temperature (Leamington; blue circles) and hypoxia (Sta E, W5, W6, W7, and W8; red circles) in the western basin. The water temperature data in 1980 is from Bartish^42^. The red crossed circles indicate the years with limited hypoxic zones (1989 and 2005) in the central basin from Zhou et al.^56^ analysis during 1987–2007 (no hypoxic zone was observed in 1996 and 2007). The red dashed lines represent the 10-year moving average (10 yr MA) based on patterns in wave power. (**b**) soluble reactive phosphorus (SRP) load: the SRP load to the water column of western basin during periods of mixing (red bars) and the SRP load in upwelled water that flows back to the central basin during calmer conditions (blue bars; negative). The dashed-red and dashed-blue lines are the 10-year moving average of the SRP load to the water column of western basin and that which flows back to the central basin, respectively, and the solid-black line is the 10-year moving average of the phosphorus load to the western basin in the upwelled water (sum of dashed-red and dashed-blue lines). The black solid circles indicate August samples that had elevated TP (total phosphorus) concentrations relative to mid-July to mid-September samples in a given year (standard deviate, ${\mathrm{Z}}_{\mathrm{TP}}$ > 1). (**c**) Average ± standard error ${\mathrm{Z}}_{\mathrm{TP}}$ measured during upwelling events (n = 11) and non-upwelling periods (n = 25) taken from 5 August–8 September between 2000 and 2018 (difference was statistically significant; see text).

We used historical records (Table S2) of long-term near-bottom water temperature (1998–2018) and dissolved oxygen (2007–2018) in the northeastern portion of the western basin of Lake Erie as well as wave observations in the western portion of the central basin (1980–2018 in Sta. NDBC 45005, Fig. 1) in August to determine the frequency of hypoxic upwelling events and the impacts of these events on the total phosphorus concentration in the northeast portion of the western basin. These analyses do not include the local hypoxia due to periods of calm and warm atmospheric conditions that may occur annually^31^ and, which are different than episodic upwelling events. Intrusion of cold hypoxic hypolimnetic water from the central basin into the western basin, following high winds from upwelling favourable directions, can cause a sudden drop (on the order of hours) in *LBT* and dissolved oxygen (DO) when the hypolimnetic water in the central basin is hypoxic^22^. The *LBT* time series in the western basin from 2017 to 2018 show that *LBT* decreased more than 3 °C in less than 12 h during upwelling events; e.g., 9–16, 18–22 and 26–31 August 2018 at Sta E (Fig. 3b) and 24–29 August 2017 at Leamington and Sta E (Fig. S10b). The records of *LBT* measured by the Ontario Ministry of Natural Resources and Forestry (MNRF) in August in Leamington Ontario between 1998 and 2018 detected 23 events of intrusion of cold water, which are consistent with upwelling (the blue symbols in Fig. 4a).

Using a simple assumption of direct-uniform flow of hypolimnetic water from the central basin, we estimate that there is high probability that hypoxic water can intrude into the western basin when the hypoxic zone of the central basin is less than ~ 12 km east of the Pelee Passage (i.e., the distance the hypolimnetic water travels with an average bottom current speed of ~ 0.10 m s^−1^^32^ over ~ 1.5 d^22^). We used Zhou et al.’s^56^ estimated spatial extent of deep-water hypoxia in the central basin from 1987 to 2007 to predict the hypoxia of the upwelled hypolimnetic water. Their results indicated a spatially limited hypoxic zone in 1989 and 2005, when upwelled waters into the western basin would not be hypoxic as denoted in Fig. 4a.

Observations from the western portion of the central basin (Sta. NDBC 45005, Fig. 1) show that hypoxic upwelling events in the western basin follow waves from the south and southwest direction with increased *WP*; e.g., 8–9, 16–17 and 25–26 August 2018 (Fig. 3a) and 22–24 August 2017 (Fig. S10a). The significant wave height and mean wave period during these periods was typically $\ge$ 0.70 m and $\ge$ 3 s, respectively (Figs. 3a and S10a). These strong waves were driven by extreme winds > 8 m s^−1^ from similar directions, which corresponds to the ~ 80th percentile of wind speeds and is greater than the sum of the average and standard deviation of the wind speed (~ 6 and 2 m s^−1^, respectively). This wind threshold is consistent with Rao et al.’s^44^ wind speed that led to upwelling, which resulted in a fish kill along the north shore of the central basin in 2012.

We used a least-square method to find a wave pattern (i.e., wave direction, duration, and power) that could be applied to predict the number of upwelling events that could be hypoxic between 1998 and 2018 based on *LBT* observations. A rapid decrease in the *LBT* at both Sta E and Leamington (12 km vs. 20 km from the Pelee Passage, respectively) occurred during events in which the average *WP* was > 0.44 kW m^−1^ (i.e., 22–24 August 2017; Fig. S10a,b). The model predicted 25 upwelling events at Leamington (dark bars in Fig. 4a) of which 23 were observed (as stated above; no data were available for 2012; blue circles in Fig. 4a) for waves from south and southwest that lasted for at least 15 h with an average wave power greater than 0.37 kW m^−1^. Of the 23 observed events, the model predicted 21 events providing a root mean square error [RMSE] of 0.20 events. We validated the model predictions using the biweekly DO measurements from MNRF cruises between 2007 and 2018, which happened to sample 17 of the 23 observed events of low *LBT*. We note, however, that two hypoxic upwelling events were also recorded outside the study period, i.e., early September; this supports the study’s focus on August. Hypoxic conditions (DO < 2 mg L^−1^) were observed for 14 of these 17 upwelling events (82%). Importantly, there was no evidence of upwelling hypoxia in 2005, which is consistent with the limited spatial extent of hypoxia in the central basin in that year^56^; unfortunately, there are no observations in the western basin for 1989.

We extended the wave power method to the wave records since 1980 to estimate the frequency of the hypoxic upwelling events into the western basin. Figure 4a shows an increasing trend in the number of predicted upwelling events; i.e., from 0.4 events year^−1^ in 1980 to > 1.6 events year^−1^ in 2018 based on a 10-year moving average. Specifically, 21 of 49 (~ 43%) upwelling events in the last four decades have occurred in the past 10 years. Thirty-two of these were strong events with *WP* > 0.44 kW m^−1^, 15 of which (~ 47%) occurred after 2009. Interestingly, this pattern in wave power (i.e., waves from south and southwest that last for > 15 h with an average *WP* > 0.37 kW m^−1^ from the historical data) was also observed in August 1980 (Fig. 4a), when the *LBT* dropped following rapid formation of a thermocline, which at the time was attributed to the upwelling of hypolimnetic water from the central basin^40,42^. These results indicate that an increase in extreme winds from south and southwest during August, over the last four decades, has resulted in more frequent upwelling from the central basin into the western basin and consequently a greater number of episodic hypoxic events in that part of Lake Erie.

The effect of upwelling on phosphorus concentrations was examined through an analysis of the water column-average total phosphorus (TP) observations from biweekly cruises conducted by the MNRF at station W5 (Fig. 1b). We examined the available data recorded between 15 July and 15 September from 2000 to 2018 (3–5 records year^−1^; 66 observations in total), which is a period in which linear patterns in TP vs. sampling date were not evident (p >  > 0.05). The z-score (standard deviate) was determined for the data within a given year (${\mathrm{Z}}_{\mathrm{TP}}=\left(\mathrm{TP}-{\mathrm{TP}}_{\mathrm{mean}}\right)/\mathrm{SD}$, where ${\mathrm{TP}}_{\mathrm{mean}}$ is the annual average of TP and SD is the standard deviation). Positive ${\mathrm{Z}}_{\mathrm{TP}}$ values (i.e., $\mathrm{TP}>{\mathrm{TP}}_{\mathrm{mean}}$) were observed in 11 cases in which the sampling occurred < 3 days after upwelling events, 30 of which were predicted in Fig. 4a. Six of these cases were among the seven occurrences of elevated TP concentrations (${\mathrm{Z}}_{\mathrm{TP}}$ > 1) observed during 5 August–8 September sampling (black solid circles in Fig. 4b). Statistical comparison revealed that the average ${\mathrm{Z}}_{\mathrm{TP}}$ was significantly higher during upwelling vs. non-upwelling samples (i.e., 0.95 ± 0.18, n = 11 vs. − 0.26 ± 0.12, n = 25; ANOVA F_1,34_ = 29.64, p < 0.001; Fig. 4c). These results indicate elevated TP in the northeastern portion of the western basin of Lake Erie following the upwelling events of high phosphorus containing hypoxic water from the central basin.

## Discussion and conclusions

The results of this study show an increase in wave power (*WP*; ~ 1.0 ± 0.6% year^−1^ between 1980 and 2018) during August in the Great Lakes. Specifically, *WP* from the south and southwest, which are along the dominant wind directions, increased by ~ 2.0 ± 0.9% year^−1^. These changes were associated with increasing significant wave heights (1.7 ± 1.5% year^−1^) driven by increasing wind stress (~ 3.0 ± 0.9% year^−1^) and are likely to have a number of effects, including higher sediment resuspension^23^. In Lake Erie, *WP* from the south and southwest, which are along the SW-NE orientation of Lake Erie and are indicative of extreme high winds favourable for hypolimnetic intrusion from the central basin into the western basin, have increased significantly by 1.4 ± 0.2% year^−1^ during August and are associated with increasing wave heights and wind speed (Fig. S1 and Table S1). It is relevant to note that the increase in wind speed during August has occurred as the seasonal (July to September) winds, which drive the typical surface and internal seiches in lakes^32^, have calmed; in contrast to August, the wind speed decreased in both July and September over this period (Fig. S11a,c). We note that the seasonal trend from 1980 to 2009 in Lake Erie Sta. NDBC 45005 (Fig. S11d; − 0.17 ± 0.04 m s^−1^ decade^−1^, R^2^ = 0.33, p < 0.001) is similar to that reported for Lake Ontario (− 0.21 ± 0.05 m s^−1^ decade^−1^, R^2^ = 0.4, p < 0.001)^3^. Whereas the Ekman transport generated by wind momentum is the driver of upwelling in this system, it is interesting to note that wind stress was only moderately predictive of upwelling events; i.e., winds from the south and southwest with an average wind stress > 0.09 Pa for at least 15 h predicts 11 out of 25 events (44%; RMSE = 1.35 events). The pattern in *WP*, however, appears to be a better proxy (not the cause) of upwelling events than wind stress ($\tau$) likely because it is a measure of the surface wind field integrated over the fetch (we note the similarity to the Wedderburn or Lake number^59^, which generally applies to lakes with single basins and was not predictive for this study). Wave power can be an indicator of water motions including significant wave height (*SWH*) and wave period (*T*_*p*_) as an outcome of winds at a local scale ($WP \propto {W}^{n > 2})$, whereas the wind stress $(\tau \propto {W}^{n = 2})$ is calculated from single wind measurement and does not include information about water motion. *WP* is also more consistent than wind stress between stations (Fig. S7c,d); the correlation between buoys Sta. NDBC 45005 and Port Stanley in Lake Erie (Fig. 1a; ~ 130 km apart) in August 2018 was *r* = 0.74 for *WP* vs. *r* = 0.62 for $\tau$. Although the *WP* appears to be an efficient tool to predict the upwelling events in the western basin of Lake Erie, a thorough understanding of these events in large water bodies such as those comprising the Great Lakes, which includes complex physical processes (e.g., highly dynamic and sloshing of thermocline, basin-scale internal waves and seiches), warrants further investigation.

Historical records show that the sudden drops in *LBT* during hypoxic upwelling events in the northeastern portion of the western basin of Lake Erie were preceded by increased *WP* in the upwelling favourable directions in the western portion of the central basin. There are a number of ecological and water quality consequences of this increased frequency of upwelled hypoxic bottom water. This includes potential impacts on the spatial distribution and population density of hypoxia-intolerant burrowing mayflies (*Hexagenia* spp.), which recolonized the basin in the mid-1990s after a 40-year absence, due to hypoxia caused by eutrophication^39^. That recovery of mayfly populations observed elsewhere in the western basin did not occur in our study region^38^. The subsequent decline in the mayfly nymph population density between 1998 and 2013 has been reported to be due to hypoxia^60^. Conversely, a more recent increase in the population density of year 1 mayfly nymphs in the western basin of Lake Erie in 2014^60^ following several years of reduced abundance is consistent with an absence of observed hypoxia that year (Fig. 4a). In terms of the present study, we observed 2–7 days during upwelling events in which the bottom DO < 1.2 mg L^−1^ (e.g., 9–16, 18–21 and 27–29 August 2018; Fig. 3b), which marks the onset of the mortality of *Hexagenia* nymphs^61^. During these events we observed 3–5 days of bottom DO < 0.4 mg L^−1^ (e.g., 10–15 and 19–21 August 2018; Fig. 3b), which is below the DO concentration that results in 100% mortality within 24 h^61^. *Hexagenia* mayflies, which are native to the western basin, are an important food resource for economically valuable Walleye and Yellow Perch and are considered a major indicator of the water quality and ecological condition of the lake^43^. Episodic hypoxia also reduces the distribution of juvenile Walleye, Yellow Perch and other species, which are components of the one of the world's largest commercial freshwater fisheries^36,37^. Hypoxia compresses fish habitat, resulting in lower fish density in the hypoxic zone, but higher fish densities near the edges of these regions^62^. This has affected the joint international management of fisheries in the western basin because data from bottom trawls, which is used to assess fish stocks and set quotas, has excluded hypoxic regions due to spatial bias since 2009^37^. The increasing frequency of episodic hypoxic events can have a significant effect on ecological processes in the region.

In addition to nutrients, water temperature and dissolved oxygen are two of the main factors controlling biogeochemical reactions in the water column and lake sediments^34^. This is another reason why hypoxic upwelling events can have profound effects on the ecosystem of lakes. There are limited data on the flux of soluble reactive phosphorus (SRP) from bottom sediments in the western basin of Lake Erie^63^. An average summertime diffusive flux of ~ 1.12 mg SRP m^−2^ day^−1^ (range 0.64–1.53 mg SRP m^−2^ day^−1^) under anaerobic conditions vs. 1.50 mg SRP m^−2^ day^−1^ under aerobic conditions using core incubation was reported in the northeastern portion of the western basin (~ 2.5 km south of Sta W7; Fig. 1b)^63^. This anaerobic SRP flux from the sediments is lower, but comparable to the aerobic SRP flux, and less than the average anaerobic value determined for western basin overall (i.e., 6.01 mg SRP m^−2^ day^−1^)^63^. The SRP flux from the sediment during hypoxic upwelling is, therefore, unlikely to contribute meaningfully to the internal loading of phosphorus in the western basin.

Conversely, the SRP load in the upwelled hypoxic hypolimnetic water from the central basin can be considerable; i.e., (~ 21 ± 18) × 10^3^ kg based on 0.035 ± 0.015 mg SRP L^−1^^49^ ×  ~ 2.0 ± 0.5 m height^22^ ×  ~ 305 ± 207 km^2^ area (an average spatial extent of hypoxic water in the western basin due to upwelling events based on numerical simulations^64^). This represents < 3.5% of the total volume of hypoxic water in the hypolimnion of the central basin (i.e., an average of ~ 5000 km^2^ area based on observations during 1987–2007^56^ × hypolimnion thickness of > 3.5 m^32,46^. It can be estimated that the SRP load in the upwelled water to the western basin was (~ 8.5 ± 7.0) × 10^3^ kg in 1980 and (~ 36 ± 30) × 10^3^ kg in 2018, based on a 10-year moving average (solid black line in Fig. 4b).

Upwelling stratification can occur in the western basin regardless of the initial conditions in the basin (i.e., thermally stratified or mixed). Nocturnal cooling, however, is not likely to mix the upwelled water because of the strength of the stratification; i.e., the large temperature gradient between the upwelled water and the water column temperature under neutral conditions. Mixing of the upwelled water is, however, predicted to occur when winds > 7 m s^−1^ causing the advective mixing of the water column due to the movement of water^31^. In this case, the SRP introduced by the upwelling events can be mixed within the water column of the western basin following the upwelling events (i.e., ~ 20% of occurrences; red bars) or it can flow back into the central basin in the hydraulic flow (i.e., ~ 80% of occurrences; blue bar) (Fig. 4b). Based on this realization, the SRP load to the water column in the western basin has increased from (~ 4.3 ± 3.6) × 10^3^ kg in 1980 to (~ 15 ± 13) × 10^3^ kg in 2018, using a 10-year moving average (red dashed lines in Fig. 4b). The latter value is ~ 24% of the monthly SRP load to the western basin between 2009 and 2013^63^. Given a water velocity of ~ 0.1 m s^−1^ and ~ 25 km of hypoxic extension in the east part of the western basin, it would take ~ 3 days for the high phosphorus water to return to the central basin. It is likely that the elevated TP concentration circulating in the water column after upwelling events, as confirmed by the field observations (Fig. 4b,c), influences water quality in the northeastern portion of the western basin. This would be in addition to the well documented nutrient enrichment from the Maumee and Detroit rivers at the western margin of the lake^63^. The increase in phosphorus due to upwelling would, therefore, represent an additional contribution to the degradation of water quality as well as the growth of HABs and nuisance algae^65^ in the northeast portion of the western basin of lake Erie.

This study shows an increasing trend in the extreme wind events and its potential impacts on the water quality (e.g., water temperature, dissolved oxygen and phosphorus) of a large lake with multiple basins. It is intriguing to suggest that the frequency of analogous wind-induced events may be increasing in other large lakes with multiple basins (e.g., basins of Lake Geneva^50^, Lake Michigan—Muskegon Lake Bay^52^, and Kempenfelt Bay of Lake Simcoe^54^), lake-rivermouth (e.g., Lake Michigan—Pere Marquette River^55^), or interconnected basins of reservoirs (e.g., Knewstubb and Natalkuz Lakes in Nechako Reservoir^51^). Clearly this is a subject that warrants further study.

## Materials and methods

### Wave power (*WP*)

The transport of energy by waves, which represents the temporal variations of energy transferred from the atmosphere to the ocean surface motion over cumulative periods of time is called wave power (*WP*). Wave power for irregular waves can be calculated as a function of the spectral energy density function $E\left(f,\theta \right)$, and the velocity at which the energy is propagating (or the group velocity) $C\left(f,h\right)$ as^16,66,67^

1 $$WP= \iint \rho gE\left(f,\theta \right) \cdot C\left(f,h\right) dfd\theta$$

where $f$ is the frequency, $\theta$ is the direction of waves, $h$ is the water depth. The group velocity is defined as $C=\frac{L}{2{T}_{p}}\left(1+\frac{2kh}{\mathrm{sinh}(2kh)}\right)$, where ${T}_{p}$ and $L$ are the wave period and wavelength, respectively, and $k=2\pi /L$ is the wavenumber. Using wave spectral parameters for an irregular sea-state, *WP* can be determined as

2 $$WP=\frac{\rho {g}^{2}}{64\pi }{ T}_{e}{ SWH}^{2},$$

where $SWH$ is the significant wave height (mean height of the highest third of the waves during typically 1 h) and ${T}_{e}$ is the energy period that can be estimated as a factor $\alpha$ = 0.538 of mean wave period (${T}_{p}$)^17,67^. Here, *WP* was calculated using hourly measurements of $SWH$ and ${T}_{p}$ in Great Lakes (Fig. 1a).

*SWH* and *T*_*P*_ can be estimated by 0.0163*X*^0.5^*W* and 0.566*X*^0.3^*W*^*0.4*^ at fetch *X* for developing waves, respectively, and by 0.0248*W*^2^ and 0.728*W* for fully developed waves, respectively^68^). Therefore, due to Eq. (2), *WP* is proportional to wind speed; i.e., $WP \propto {W}^{2.4}$ and $\propto {W}^{5}$ for developing and fully developed waves, respectively. We used field measurements of *SWH* and *TP* for calculation of *WP*.

Wind stress ($\tau )$ is calculated by

3 $$\tau ={C}_{d}{\rho }_{air}{W}^{2},$$

where ${\rho }_{air}$= 1.22 kg m^−3^ is the density of air and ${C}_{d}=$ 0.0012 is the drag coefficient^57^, and $W$ is measured 10 m above the surface.

### Mutual information (MI)

Here MI was calculated following Hoyos et al.^69^ to measure the dependence between lake surface temperature (*LST*_*w*_) and wave power *WP*_*w*_ in Lake Erie (Sta. NDBC 45005) during August between 1980 and 2018 (Fig. S8). Based on information theory, mutual information is a measure of the information two variables $X$ and $Y$ share, which quantifies the level of their statistical independence^16,68^. Specifically, MI quantifies the difference between the joint distribution of two variables and the product of their marginal distributions, which are equal for independent variables. The entropy of $X$ for a random event $x$ is defined as

4 $$\mathrm{H}\left(X\right)=\sum p\left(x\right){log}_{2}p\left(x\right),$$

where $p\left(x\right)$ is the probability. The joint entropy of $X$ and $Y$ is given by

5 $$\mathrm{H}\left(X,Y\right)=\sum g\left(x,y\right){log}_{2}g\left(x,y\right)$$

where $g\left(x,y\right)$ is the joint probability of $x$ and $y$. The MI is calculated as

6 $$\mathrm{MI}\left(X,Y\right)=\sum g\left(x,y\right){log}_{2}\frac{g\left(x,y\right)}{p\left(x\right)q\left(y\right)}=H\left(X\right)+H\left(Y\right)-H(X,Y)$$

Therefore, for two independent variables $X$ and $Y$ that do not contain any information about each other, the total entropy of their system (i.e., $\mathrm{H}\left(X,Y\right)$) would be equal to the sum of their entropies (i.e., $H\left(X\right)+H\left(Y\right)$) resulting in $\mathrm{MI}\left(X,Y\right)$ = 0.

In Lake Erie, the product of the marginal distributions of *LST*_*w*_ and *WP*_*w*_ (Fig. S8c) differs from their joint distribution (Fig. S8d), which can be quantified as MI of 1.6 for entropies of 3.2 in *WP*_*w*_ and 2.8 in *LST*_*w*_. In addition, their joint distribution shows that higher values are located along the diagonal confirming that the changes in values of *LST* are closely related to changes in wave power (Fig. S8d). Our implementation was validated against global wave power and sea surface temperature presented in Reguero et al.^16^.

### Measurements

We used the longest available buoy data in Great Lakes since 1980 that include weather, wave, and surface water temperature (*LST*) (Fig. 1a). The data in the western basin of Lake Erie include time series of bottom water temperature (*LBT*), dissolved oxygen, and currents in 2017 (23 to 31 August)^22^ and 2018 (1 to 31 August) at Sta E, time series of *LBT* in Leamington from the Ontario Ministry of Natural Resources and Forestry (MNRF) since 1998, and historical limnological records (including bottom water oxygen, temperature, total phosphorus) from biweekly cruises conducted in August by MNRF at stations W5, W6, W7, and W8 (see Table S2). The total phosphorus were measured from the water samples collected at 1 m below surface and 2 m above bottom (or 1 m above the thermocline when stratified) and halfway between, which were combined and analyzed using standard methods (4500-P^70^).

### Statistical analysis

Linear regression and ANOVA (analysis of variation) techniques were applied to data that were normally distributed and exhibited homogeneity of variation as examined under Shapiro–Wilks and Levene’s tests, respectively.

## Supplementary Information

Supplementary Information.

## Data Availability

All the data and codes in this study can be obtained from the corresponding author upon the request of the reviewers and will be available online after publication.
