# Supplementary material for: Increases in Great Lake winds and extreme events facilitate interbasin coupling and reduce water quality in Lake Erie
Source: Sci Rep. 2021 Mar 11;11:5733. doi: 10.1038/s41598-021-84961-9 (PMC7970988; doi:10.1038/s41598-021-84961-9)
Supplement: Supplementary file 1 — Supplementary Information. [file 41598_2021_84961_MOESM1_ESM.docx]

**Supplementary Materials for:**

**Increases in Great Lake winds and extreme events facilitate interbasin coupling and reduce water quality in Lake Erie**

Aidin Jabbari^1†^, Josef D. Ackerman^1*^, Leon Boegman^2^, and Yingming Zhao^3^

1. *Physical Ecology Laboratory, Department of Integrative Biology, University of Guelph, Guelph, ON, Canada*

2. *Environmental Fluid Dynamics Laboratory, Department of Civil Engineering, Queen’s University, Kingston, ON, Canada*

3. *Ontario Ministry of Natural Resources and Forestry, Aquatic Research and Monitoring Section, Wheatley, ON, Canada*

^*^Corresponding Author: [ackerman@uoguelph.ca](mailto:ackerman@uoguelph.ca), Tel: 519-824-4120 x 58268

^†^Present address: Biogeochemistry and Earth System Modelling, Department of Geoscience,

Environment and Society, niversité Libre de Bruxelles, Bruxelles, Belgium

| **Table S1 \| Results of the linear regression of time series of historical lake water temperature, wind, and wave observations in Great Lakes during August** | | | | | | | | |  |  |  |  |  |
| --- | --- | --- | --- | --- | --- | --- | --- | --- | --- | --- | --- | --- | --- |
| *Location* | *Variable*  (all  directions) | Slope ± SE | R^2^ | p-value | *Variable_w_* (during winds from south and southwest) | Slope ± SE | R^2^ | p-value |  |  |  |  |  |
| Lake Erie  (Sta. NDBC 45005;  41.677^∘^N, 82.398^∘^W; 9.8 m depth)  1980 to 2018, *n* = 39 | $LST$ | 0.16 ± 0.15 | 0.03 | 0.29 | ${LST}_{w}$ | 0.16 ± 0.15 | 0.03 | 0.28 |  |  |  |  |  |
|  | $W$ | 0.51 ± 0.09 | 0.44 | **<0.001** | $W_{w}$ | 0.61 ± 0.11 | 0.46 | **<0.001** |  |  |  |  |  |
|  | $\tau$ | 0.01 ± 0.001 | 0.48 | **<0.001** | $\tau_{w}$ | 0.01 ± 0.002 | 0.49 | **<0.001** |  |  |  |  |  |
|  | $SWH$ | 0.02 ± 0.009 | 0.14 | **0.019** | ${SWH}_{w}$ | 0.03 ± 0.008 | 0.23 | **0.002** |  |  |  |  |  |
|  | $WP$ | 0.02 ± 0.02 | 0.02 | 0.38 | ${WP}_{w}$ | 0.02 ± 0.01 | 0.14 | **0.019** |  |  |  |  |  |
| Lake Ontario  (Prince Edward Pt;  43.780^∘^N, 76.870^∘^W;  68 m depth)  1990 to 2018, *n* = 29 | $LST$ | 0.48 ± 0.18 | 0.20 | **0.015** | ${LST}_{w}$ | 0.49 ± 0.21 | 0.15 | **0.03** |  |  |  |  |  |
|  | $W$ | 0.25 ± 0.08 | 0.28 | **0.003** | $W_{w}$ | 0.36 ± 0.09 | 0.34 | **<0.001** |  |  |  |  |  |
|  | $\tau$ | 0.005 ± 0.0015 | 0.27 | **0.003** | $\tau_{w}$ | 0.006 ± 0.002 | 0.32 | **0.001** |  |  |  |  |  |
|  | $SWH$ | 0.077 ± 0.015 | 0.5 | **<0.001** | ${SWH}_{w}$ | 0.09 ± 0.014 | 0.63 | **<0.001** |  |  |  |  |  |
|  | $WP$ | 0.052 ± 0.020 | 0.21 | **0.013** | ${WP}_{w}$ | 0.076 ± 0.021 | 0.31 | **0.0017** |  |  |  |  |  |
| Lake Huron  (NDBC45008;  44.283^∘^N, 82.416^∘^W;  54.3 m depth)  1981 to 2018, *n* = 38 | $LST$ | 0.49 ± 0.2 | 0.15 | **0.017** | ${LST}_{w}$ | 0.48 ± 0.21 | 0.13 | **0.03** |  |  |  |  |  |
|  | $W$ | 0.22 ± 0.08 | 0.17 | **0.011** | $W_{w}$ | 0.3 ± 0.08 | 0.28 | **<0.001** |  |  |  |  |  |
|  | $\tau$ | 0.003 ± 0.0015 | 0.15 | **0.018** | $\tau_{w}$ | 0.005 ± 0.001 | 0.26 | **0.001** |  |  |  |  |  |
|  | $SWH$ | 0.011 ± 0.013 | 0.02 | 0.39 | ${SWH}_{w}$ | 0.027 ± 0.011 | 0.16 | **0.014** |  |  |  |  |  |
|  | $WP$ | 0.022 ± 0.019 | 0.04 | 0.24 | ${WP}_{w}$ | 0.039 ± 0.015 | 0.15 | **0.02** |  |  |  |  |  |
| Lake Michigan  (NDBC45007;  42.674^∘^N, 87.026^∘^W;  159.1 m depth)  1981 to 2018, *n* = 38 | $LST$ | 0.29 ± 0.21 | 0.05 | 0.17 | ${LST}_{w}$ | 0.28 ± 0.21 | 0.04 | 0.20 |  |  |  |  |  |
|  | $W$ | 0.20 ± 0.075 | 0.17 | **0.01** | $W_{w}$ | 0.25 ± 0.01 | 0.15 | **0.016** |  |  |  |  |  |
|  | $\tau$ | 0.003 ± 0.001 | 0.16 | **0.016** | $\tau_{w}$ | 0.004 ± 0.001 | 0.16 | **0.013** |  |  |  |  |  |
|  | $SWH$ | 0.024 ± 0.0.11 | 0.11 | **0.04** | ${SWH}_{w}$ | 0.028 ± 0.015 | 0.1 | 0.06 |  |  |  |  |  |
|  | $WP$ | 0.032 ± 0.026 | 0.04 | 0.22 | ${WP}_{w}$ | 0.077 ± 0.017 | 0.37 | **<0.001** |  |  |  |  |  |
| Lake Superior  (NDBC45001;  247 m depth;  48.061^∘^N, 87.793^∘^W)  1981 to 2018, *n* = 38 | $LST$ | 0.55 ± 0.45 | 0.04 | 0.22 | ${LST}_{w}$ | 0.52 ± 0.47 | 0.03 | 0.20 |  |  |  |  |  |
|  | $W$ | 0.30 ± 0.09 | 0.23 | **0.002** | $W_{w}$ | 0.26 ± 0.10 | 0.15 | **0.016** |  |  |  |  |  |
|  | $\tau$ | 0.004 ± 0.0014 | 0.21 | **0.003** | $\tau_{w}$ | 0.004 ± 0.0016 | 0.14 | **0.022** |  |  |  |  |  |
|  | $SWH$ | 0.018 ± 0.014 | 0.04 | 0.20 | ${SWH}_{w}$ | 0.038 ± 0.018 | 0.11 | **0.043** |  |  |  |  |  |
|  | $WP$ | 0.054 ± 0.029 | 0.10 | 0.07 | ${WP}_{w}$ | 0.081 ± 0.026 | 0.21 | **0.004** |  |  |  |  |  |
| Average over  Great Lakes ± standard deviation | $LST$ | 0.39 ± 0.16 |  |  | ${LST}_{w}$ | 0.39 ± 0.16 |  |  |  |  |  |  |  |
|  | $W$ | 0.30 ± 0.12 |  |  | $W_{w}$ | 0.36 ± 0.14 |  |  |  |  |  |  |  |
|  | $\tau$ | 0.005 ± 0.003 |  |  | $\tau_{w}$ | 0.006 ± 0.002 |  |  |  |  |  |  |  |
|  | $SWH$ | 0.03 ± 0.02 |  |  | ${SWH}_{w}$ | 0.043 ± 0.026 |  |  |  |  |  |  |  |
|  | $WP$ | 0.036 ± 0.016 |  |  | ${WP}_{w}$ | 0.059 ± 0.027 |  |  |  |  |  |  |  |
| Regression results of lake surface temperature anomaly ($LST$; ^o^C decade^-1^), 10-m wind speed ($W$; m s^-1^ decade^-1^), wind stress ($\tau$; Pa decade^-1^), significant wave height ($SWH$; m decade^-1^), and wave power ($WP$; kW m^-1^ decade^-1^) during August. Data are from all of August (left side) and from August during periods of wind from south and southwest (with subscript “*w*”; right side). Here SE = standard error; R^2^ = coefficient of determination; p-value = probability that the slope is equal to zero. Statistically significant relationship (i.e. p-value < 0.05) are provided in bold font. | | | | | | | | |  |  |  |  | ${WP}_{w}$ |

| **Table S2 \|** **Station locations and instruments in Lake Erie** | | | | | | | |
| --- | --- | --- | --- | --- | --- | --- | --- |
| Station | Measurement  Period | Latitude, Longitude | Depth  (m) | Instruments | Sampling Frequency | Height Above  Bottom (m) | Source |
| Sta. E | 2017 (Aug 23-30)  2018 (Aug 1-30) | 41.9398^∘^N, 82.5527^∘^W | 10.6 | DO (RBR-1060)  *LBT* (Tidbit; Onset) | 5 min  30 min | 1  1 | Authors  (University of Guelph) |
| Leamington | 1998 to 2018 except 2012  (Aug 1-30) | 42.01567^∘^N, 82.58185^∘^W | 4.1 | *LBT* (Tidbit; Onset) | 30 min | 0.5 | MNRF |
| W5 | 2007 to 2018 except 2012  (Aug 1-30) | 41.8832^∘^N, 82.613^∘^W | 10.7 | DO, TP, and *LBT* (Hydrolab/YSI EXO2) | biweekly | < 1 | MNRF |
| W6 | 2007 to 2018 except 2012  (Aug 1-30) | 41.8545^∘^N, 82.7623^∘^W | 11.1 | DO and *LBT* (Hydrolab/YSI EXO2) | biweekly | < 1 | MNRF |
| W7 | 2007 to 2018 except 2012  (Aug 1-30) | 41.9907^∘^N, 82.7633^∘^W | 9.2 | DO and *LBT* (Hydrolab/YSI EXO2) | biweekly | < 1 | MNRF |
| W8 | 2007 to 2018 except 2012  (Aug 1-30) | 41.9865^∘^N, 82.5758^∘^W | 9.6 | DO and *LBT* (Hydrolab/YSI EXO2) | biweekly | < 1 | MNRF |
| Sta. NDBC 45005 | 1980 to 2018  (Aug 1-30) | 41.677^∘^N, 82.398^∘^W | 9.8 | *LST* and wave | 1 hr | 9.8 | NDBC |
| Port Stanley | 1990 to 2018  (Aug 1-30) | 42.460^∘^N, 81.220^∘^W | 22 | wave | 1 hr | 22 | Environment and Climate Change Canada |
|  | | | | | | | |

| August | August-during winds  from south and southwest |
| --- | --- |
|  | |
| **Fig. S1 \| Historical patterns in atmosphere and lake water conditions in Lake Erie (Sta. NDBC 45005).** Average lake surface temperature (*LST*; **a** and **b**), wind speed at 10-m height (*W*; **c** and **d**), wind stress (*τ*; **e** and **f**), significant wave height (*SWH*; **g** and **h**), and wave power (*WP*; **i** and **j**). Data presented are for all of August (left side) and from August during periods of upwelling favorable wind directions (i.e. from south and southwest; with subscript “*w*”) (right side). The red line and the black dashed lines represent the linear regression and the 10-year moving average, respectively. Statistical results are provided in Table S1. | |
| August | August-during winds  from south and southwest |
|  | |
| **Fig. S2 \| Historical patterns in atmosphere and lake water conditions in Lake Ontario.** Average lake surface temperature (*LST*; **a** and **b**), wind speed at 10-m height (*W*; **c** and **d**), wind stress (*τ*; **e** and **f**), significant wave height (*SWH*; **g** and **h**), and wave power (*WP*; **i** and **j**). Data presented are for all of August (left side) and from August during winds from south and southwest; with subscript “*w*”) (right side). The red line and the black dashed lines represent the linear regression and the 10-year moving average, respectively. Statistical results are provided in Table S1. | |
| August | August-during winds  from south and southwest |
|  | |
| **Fig. S3 \| Historical patterns in atmosphere and lake water conditions in Lake Huron.** Average lake surface temperature (*LST*; **a** and **b**), wind speed at 10-m height (*W*; **c** and **d**), wind stress (*τ*; **e** and **f**), significant wave height (*SWH*; **g** and **h**), and wave power (*WP*; **i** and **j**). Data presented are for all of August (left side) and from August during winds from south and southwest; with subscript “*w*”) (right side). The red line and the black dashed lines represent the linear regression and the 10-year moving average, respectively. Statistical results are provided in Table S1. | |
| August | August-during winds  from south and southwest |
|  | |
| **Fig. S4 \| Historical patterns in atmosphere and lake water conditions in Lake Michigan.** Average lake surface temperature (*LST*; **a** and **b**), wind speed at 10-m height (*W*; **c** and **d**), wind stress (*τ*; **e** and **f**), significant wave height (*SWH*; **g** and **h**), and wave power (*WP*; **i** and **j**). Data presented are for all of August (left side) and from August during winds from south and southwest; with subscript “*w*”) (right side). The red line and the black dashed lines represent the linear regression and the 10-year moving average, respectively. Statistical results are provided in Table S1. | |
| August | August-during winds  from south and southwest |
|  | |
| **Fig. S5 \| Historical patterns in atmosphere and lake water conditions in Lake Superior.** Average lake surface temperature (*LST*; **a** and **b**), wind speed at 10-m height (*W*; **c** and **d**), wind stress (*τ*; **e** and **f**), significant wave height (*SWH*; **g** and **h**), and wave power (*WP*; **i** and **j**). Data presented are for all of August (left side) and from August during winds from south and southwest; with subscript “*w*”) (right side). The red line and the black dashed lines represent the linear regression and the 10-year moving average, respectively. Statistical results are provided in Table S1. | |

|  |
| --- |
| **Fig. S6 \| Historical patterns in the air temperature.** Average air temperature during August ($Tair$; **a**) and during the south and southwest winds in the August (${Tair}_{w}$; **b**) recorded in Great Lakes (Fig. 1a). The dashed lines represent the 10-year moving average. |

|  |
| --- |
| **Fig. S7 \| Wind and wave records at different stations in Lake Erie.** Wind direction (**a**), wind speed (*W*; **b**), wind stress ($\tau$; **c**), and wave power (*WP*; **d**) in Sta. NDBC 45005 (black) and Port Stanley (red) in Lake Erie (Fig. 1**a**) in August 2018. The power (**c**) and stress (**d**) of the waves and winds, respectively, from south and southwest (i.e., favorable for upwelling) are positive. |

**Fig. S8 | Statistical dependency of wave power and water surface temperature.** Statistical analysis of the dependency of wave power (*W*P) on the lake surface temperature (*LST*) anomaly during the waves from the south and southwest in Lake Erie recorded in Sta. NDBC 45005 during August 1980-2018. Probability density functions (PDFs) of (**a**) *LST* anomaly and (**b**) *WP*; the joint distribution of *WP* and *LST* anomaly (**c**) and the product of their marginal distributions (**d**) (scaled to the values in **c**). The red lines in **a** and **b** are fitted to the data in the bar plots.

|  |
| --- |
| **Fig. S9 \| Water temperature and wave power in Great Lakes associated with atmospheric phenomena.** Lake surface temperature anomaly (*LST*; **a**), Atlantic Multidecadal Oscillation (AMO; **b**), wave power (*WP_w_*; **c**), and Multivariate El Niño/Southern Oscillation index (MEI; **d**). (**a**) and (**c**) are in August during winds from the south and southwest. The black dashed lines in **c** show year corresponding to strong El Niño events (MEI greater than 1.5). The dashed lines in (**a**) and (**b**) represent the 10-year moving average. |
|  |

| 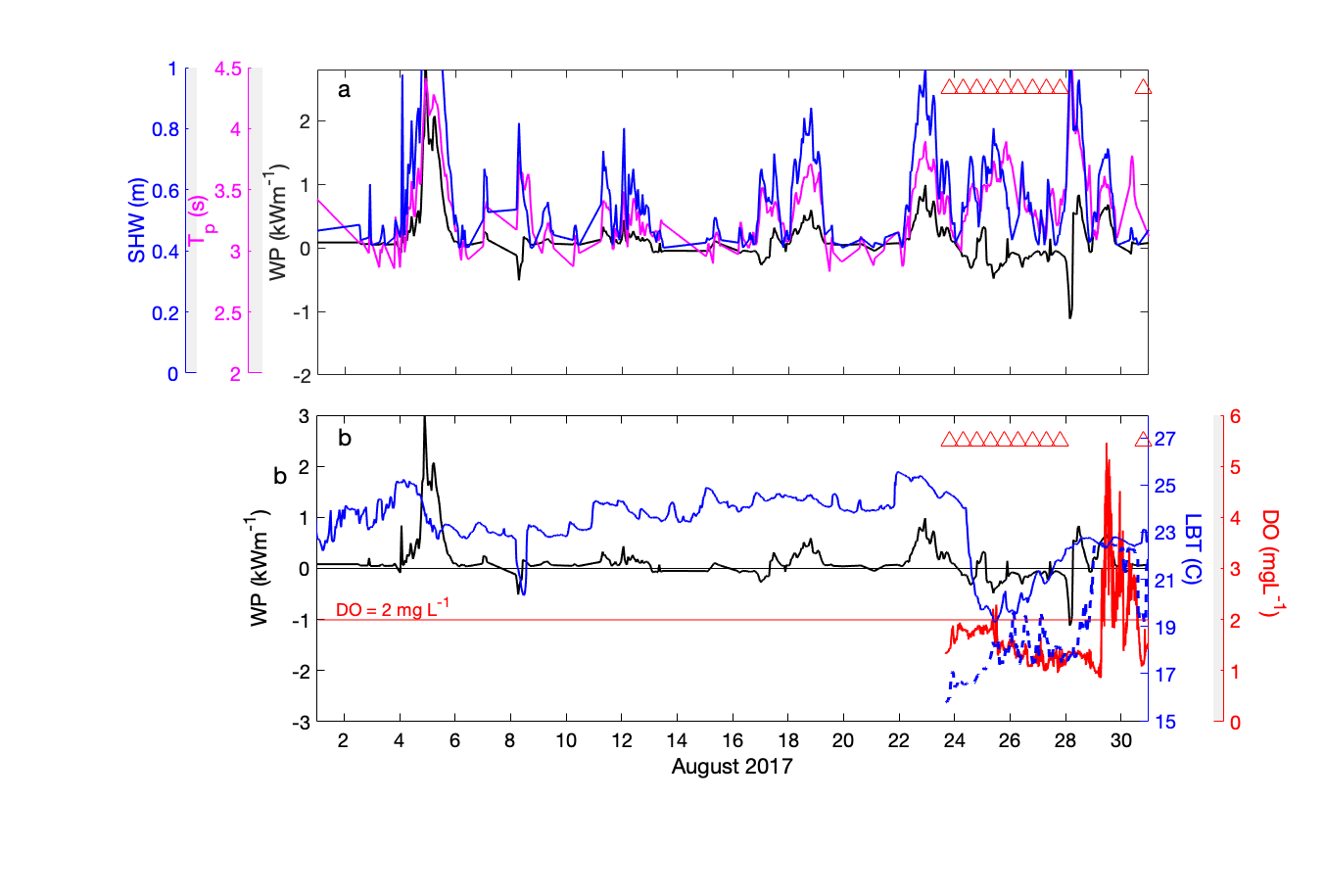 |
| --- |
| **Fig. S10 \| Wave power and bottom water temperature during August 2017 in the western basin of Lake Erie. a**, Time series of wave power (*WP*; black line), wave period (*T_p_*; magenta), and significant wave height (*SWH*; blue) recorded at Sta. NDBC 45005. **b**, Time series of dissolved oxygen (DO; red) and water temperature (*LBT*; blue dashed-line) in Sta. E at 1 m above the bed and bottom water temperature in Leamington (blue solid-line) in August 2017. Measurements in Sta E are from 23 to 31 August. The red triangles represent the observed hypoxic events in the western basin of Lake Erie. The wave power of the waves from south and southwest (i.e. favorable for upwelling) are positive preceding upwelling. |

| 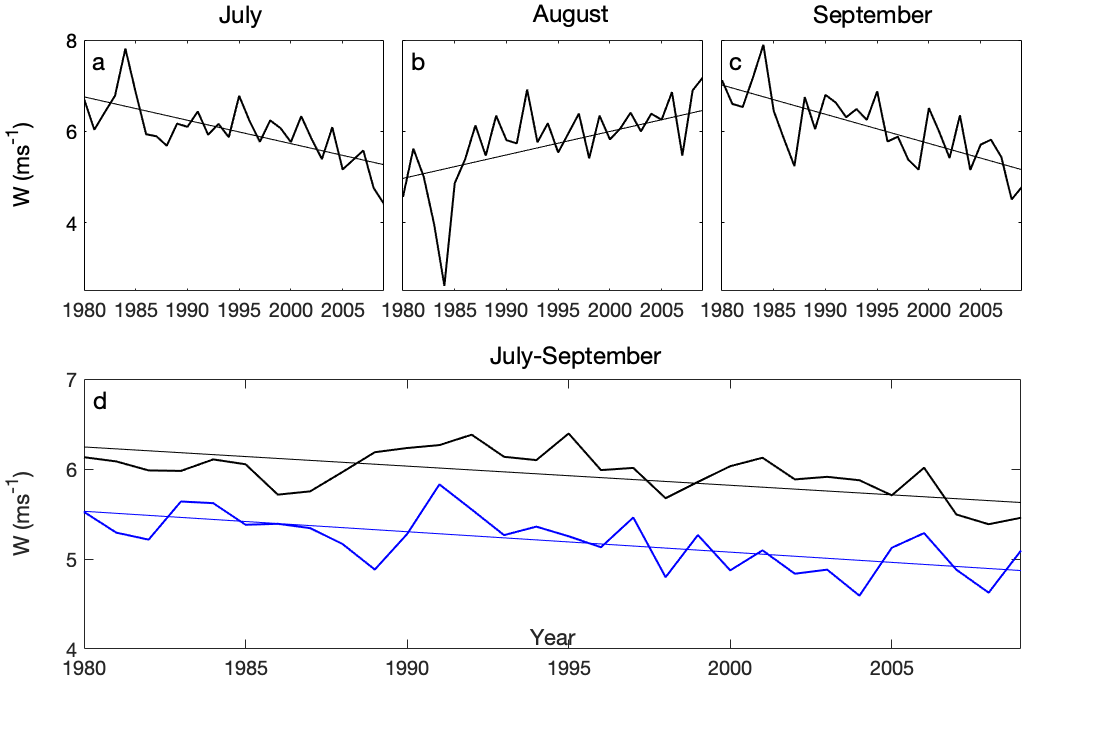 |
| --- |
| **Fig. S11 \|** Wind speed patterns during (a) July, (b) August, and (c) September in Lake Erie Sta. NDBC 45005 and (d) July-September in Lake Erie Sta. NDBC 45005 (black) and Lake Ontario (from Huang et al., 2012 (3); blue). The straight lines show the linear regression. |
